# Clinico-epidemiological characteristics of adolescents and young adults living with HIV in Ghana

**DOI:** 10.11604/pamj.2024.48.54.37911

**Published:** 2024-06-11

**Authors:** Vincent Ganu, Oluwakemi Oladele, Emmanuella Amankwa, Rafiq Okine, Peter Puplampu

**Affiliations:** 1 Department of Internal Medicine, Korle Bu Teaching Hospital, Accra, Ghana,; 2 World Health Organization Ghana Office, Accra, Ghana; 3 Department of Medicine and Therapeutics, University of Ghana Medical School, College of Health Sciences, Accra, Ghana

**Keywords:** Adolescent, young adults, human immunodeficiency virus, vertical transmission, antiretroviral therapy, orphan

## Abstract

**Introduction:**

sub-Saharan Africa is experiencing a boom in the number of adolescents and young adults living with HIV (AYALHIV). Existing HIV intervention programs are mainly for children and adults living with HIV, with little attention paid to AYALHIV. Characterizing this population is necessary for planning, and designing, AYALHIV-centered HIV intervention programs.

**Methods:**

a retrospective single-center, hospital-based chart review was conducted at the largest HIV clinic in Ghana. We examined routinely collected data for AYALHIV (aged 10-24 years) on antiretroviral therapy (ART) for at least 1 year and in active care from 1^st^ January to 31^st^ December 2019. Data was collected using a structured data extraction form. The Chi-square and the Student´s t-test were used to compare characteristics between adolescents and young adults.

**Results:**

of 252 AYALHIV, 68% (172/252) were adolescents with a median age of 17 years (IQR 13-19); 32% were young adults with a median age of 22 years (IQR: 20-24). Most (56.7% (143/252)) AYALHIV were female. Almost 40% were orphans. Eighty-six percent of AYALHIV had HIV type I infection. The commonest mode of HIV acquisition among adolescents was vertical transmission (70.5%) and that among young adults was via unprotected sex (31.3%). Eighty-eight percent (88%) of AYALHIV were on non-nucleoside reverse transcriptase inhibitors-based regimen. The viral suppression rate among AYALHIV was 78%.

**Conclusion:**

the study shows there is a growing population of AYALHIV most of which are adolescents. About two-fifths were orphans. Policymakers and HIV programs should ensure AYALHIV-centred interventions are developed for this vulnerable population.

## Introduction

Adolescents and young adults living with HIV (AYALHIV) are increasing in number globally [1]. More than two-thirds of them are in sub-Saharan Africa (SSA), with 390,000 living in West and Central Africa [1]. This is due mainly to the advent of antiretroviral drugs, resulting in increased survival of perinatally infected children. Infection through unprotected sexual intercourse among HIV-uninfected youth also partly contributes to this [2-4]. The complex developmental phase of this population exacerbates the challenges of being HIV-positive and the need for ARV adherence [5,6]. Thus, treatment outcomes are usually poorer among AYALHIV compared to adults [7,8]. Due to youthful exuberance, this population is more likely to engage in unprotected sexual intercourse, and thus, may be potential sources of new human immunodeficiency virus (HIV) infections. Again, due to their complex and unique physiological, psychological, and social needs, AYALHIV requires tailored health service interventions to keep them from being sources of new HIV infections [6, 9,10].

The continuous fight against HIV has been effectively implemented on a global scale through declarations, targets, goals, and commitments by international organizations, world leaders, and governments [11]. Despite the inclusion of young people in these targets, disaggregated data on adolescents and young adults is sparse in national and global reports [12]. This limited data contributes to challenges in assessing and monitoring progress in HIV programs for AYALHIV [11]. Compared to infants and adults, data specific to AYALHIV and their HIV care are limited [11], especially in West Africa. Data regarding coverage, uptake, and quality of services, along with comprehensive information about the adolescents and young adults who access these services, will provide significant insights into the needs of AYALHIV. It is important for national HIV programs to get data specific to AYALHIV to aid in effective and efficient planning and execution of AYALHIV-specific program implementation activities [13,14]. Thus, there is a need to characterize the AYALHIV population as part of evidence-based data to inform HIV programming and improve public resource allocation and existing services. Also, this can be used to inform or guide policy on possible future interventions for AYALHIV [14,15]. This study, therefore, sought to describe the clinical characteristics of the AYALHIV population at a tertiary hospital in Accra, Ghana.

## Methods

**Study design and site:** a retrospective single-center, hospital-based chart review on AYALHIV accessing HIV services from 1^st^ January - 31^st^ December 2019 was conducted at the HIV clinic of the Department of Medicine and Therapeutics of the Korle Bu Teaching Hospital in Ghana. This was part of a larger study assessing medication adherence challenges among AYALHIV. The Korle Bu Teaching Hospital is the main tertiary hospital that serves the whole of southern part of Ghana. The HIV clinic runs a special outpatient clinic for AYALHIV only on Thursdays, with an average attendance of 20 patients. This special clinic for AYALHIV is the only one in the Greater Accra region which is one of the high HIV burden regions in Ghana and focuses only on patients aged 10-24 years. This clinic starts as early as 6 am to ensure the AYALHIV can be attended to quickly and then be able to go to school as well to avoid missing school days due to clinic visits. Combination antiretroviral therapy (cART) is provided at the HIV clinic for all AYALHIV as the “Treat All” agenda is being implemented. The first line cART used during the period under review was the non-nucleoside reverse transcriptase inhibitor (NNRTI)- based regimen, consisting of two nucleoside reverse transcriptase inhibitors (NRTIs) plus one NNRTI (either Efavirenz or Nevirapine) as per the national treatment guidelines [16]. The second line cART was a ritonavir-boosted protease inhibitor (PI)- based regimen, comprising two NRTIs and one ritonavir-boosted PI (either Lopinavir/r or Atazanavir/r) [16].

**Study participants:** all patients aged 10 to 24 years who were active in care and on ART for at least 1 year at the HIV clinic of the Korle Bu Teaching Hospital as of the 31^st^ December 2019, were included in the study.

**Data collection:** structured data extraction forms were used to retrieve information from the clinical files of AYALHIV at the HIV clinic. Data extracted related to socio-demographic characteristics such as sex, age, religion, educational status, and marital status. Clinical characteristics obtained included mode of transmission, HIV type, antiretroviral regimen, immunological and virological data, orphan status, and HIV status of parents. Data were collected by trained research assistants, who were monitored by study investigators. The extraction forms were reviewed daily to ensure completeness, validate data, and also improve data quality.

**Definitions:** adolescent: any patient between the ages of 10 - 19 years who accessed services at the HIV clinic [17]. Young adult: any patient aged 20-24 years who accessed services at the AYALHIV clinic [17]. Viral suppression: HIV viral load levels that were less than 1000 copies/ml as outlined in the national treatment guidelines [16].

**Data analysis:** data obtained from the study were entered and analyzed using STATA version 13.0. Data were entered as continuous or coded categorical variables. Categorical variables were summarized as frequencies and percentages, and continuous variables such as age, were summarized as medians, interquartile ranges, means, and standard deviations. The chi-square or Fisher´s exact or the Student´s t-test was used based on the nature of the variable to compare relevant variables between males and females. The Mann-Whitney U test was used for the comparison of the median for variables not normally distributed. The significance level was set at 95%.

**Ethical issues:** ethical approval was obtained from the Scientific and Technical Committee as well as the Institutional Review Board of the Korle-Bu Teaching Hospital with approval identification number KBTH-STC/IRB/000183/2020. Permission was also obtained from the head of the HIV clinic before accessing data from patients´ files. All data collected were treated confidentially by removing all personal identifiers from the data extraction forms and assigning unique study codes to each AYALHIV.

## Results

**Sociodemographic characteristics:** a total of 252 AYALHIV were actively in care, with approximately 68% (172/252) being adolescents. The majority (56.7% (143/252)) of AYALHIV were female. The adolescents had a median age of 17 (IQR: 13-19) years, while the young adults had a median age of 22 (IQR: 20-24) years. About 98% of AYALHIV had at least primary-level education (Table 1). Almost 38% had lost their mothers, while 33.7% had no fathers and 15% (37/252) had both parents dead (Table 1).

**Table 1:** socio-demographic characteristics of AYALHIV at the HIV clinic of the KBTH, Accra, Ghana, December, 2019

	Total	Adolescents n=172	Young adults n = 80
Characteristic	Frequency (%)		
**Gender (n=252)**			
Male	109 (43.3)	77 (44.8)	32 (40)
Female	143 (56.7)	95 (55.2)	48 (60)
**Educational level (n=248)**			
Primary	37 (14.9)	32 (18.8)	5 (6.4)
JHS	93 (37.5)	74 (43.5)	19 (24.4)
SHS	93 (37.5)	60 (35.3)	33 (42.3)
Tertiary	25 (10.1)	4 (2.4)	21 (26.9)
**Religion (n=248)**			
Christian	220 (88.7)	149 (88.2)	71 (89.9)
Muslim	28 (11.3)	20 (11.8)	8 (10.1)
**Marital status (n=250)**			
Single	244 (97.6)	168 (98.8)	76 (95)
Married	4 (1.6)	2 (1.2)	2 (2.5)
Co-habiting	2 (0.8)	-	2 (2.5)
**Orphan status**			
**Mother alive (n=215)**			
Yes	116 (53.9)	78 (50.7)	38 (62.3)
No	81 (37.7)	66 (42.9)	15 (24.6)
Unknown	18 (8.4)	10 (6.5)	8 (13.1)
**Father alive (n=211)**			
Yes	107 (50.7)	80 (52.3)	27 (46.6)
No	71 (33.7)	50 (32.7)	21 (36.2)
Unknown	33 (15.6)	23 (15.0)	10 (17.2)
**Both parents dead (n=37)**	**37 (14.6)**	**27 (15.7)**	**10 (12.5%)**

AYALHIV: adolescents and young adults living with HIV; HIV: human immunodeficiency virus; KBTH: Korle Bu Teaching Hospital; JHS: junior high school; SHS: senior high school

**Clinical characteristics:** the majority (97.2%) of AYALHIV had HIV type I only. None of them had HIV type II only (Table 2).

**Table 2:** HIV typing of AYALHIV and their parental HIV status at the HIV clinic of the KBTH, Accra, Ghana, December 2019

	Adolescents n=172	Young adults n=80
Clinical history	Frequency (%)	
**H1V type (n=252)**		
I	168 (97.7)	77 (96.3)
II	0 (0.0)	0 (0.0)
I and II	4 (2.3)	3 (3.8)
**Mother's status (n=186)**		
Reactive	122 (89.0)	24 (48.9)
Non-reactive	3 (2.2)	21 (42.9)
Unknown	12 (8.8)	4 (8.2)
**Father's status (n=176)**		
Reactive	63 (49.6)	13 (26.5)
Non-reactive	25 (19.7)	25 (51.0)
Unknown	39 (30.7)	11 (22.5)

AYALHIV: adolescents and young adults living with HIV; HIV: human immunodeficiency virus; KBTH: Korle Bu Teaching Hospital

**Mode of acquisition of HIV:** most (88.1% (222/252)) of AYALHIV acquired the HIV infection through mother-to-child transmission and 10.7% (27/252) acquired the infection through unprotected sex and the rest (1.2% (3/252)) through rape. Among the adolescents, 98.3% (169/172) acquired HIV via MTCT whilst among the young adults, 66.3% (53/80) acquired HIV via MTCT and the rest through sex.

**Antiretroviral therapy history:** most of the AYALHIV (88.1% (222/252)) were on first-line ART (NNRTI-based regimen) with the rest on second-line ART (PI-based regimen) (Table 3). The majority of adolescents (85.5%) and young adults (93.8%) were on first-line ART (NNRTI-based regimen) (Table 3).

**Table 3:** antiretroviral treatment among AYALHIV at the HIV clinic of the KBTH, Accra, Ghana, December 2019

Antiretroviral drugs	Overall (%) (n=252)	Adolescents (%) (n=172)	Young adults (%) (n=80)
First line ART (NNRTI based regimen)*	222 (88.1)	147 (85.5)	75 (93.8)
Second line ART (PI based regimen) ∞	30 (11.9)	25 (14.5)	5 (6.2)

Efavirenz or Nevirapine plus any 2 NRTIs; ∞ Lopinavir/r or Atazanavir/r plus any 2 NRTIs; NNRTIs: non-nucleoside reverse transcriptase inhibitor; AYALHIV: adolescents and young adults living with HIV; HIV: human immunodeficiency virus; KBTH: Korle Bu Teaching Hospital

**Laboratory characteristics:** the median hemoglobin among AYALHIV was 10.5 g/dl. The median hemoglobin for adolescents and young adults was 10.3 g/dl and 11.5 g/dl respectively (Table 4). Young adults had higher average creatinine levels (68 micromol/l) than adolescents (49 micromol/l).

**Table 4:** laboratory characteristics of AYALHIV at the HIV clinic of the KBTH, Accra, Ghana, December 2019

	Median (LQ, UQ)			
Laboratory parameters	All participants (n=252)	Adolescents (n=172)	Young adults (n=80)	p-value
Hemoglobin (g/dl)	10.5	10.3	11.5	<0.0001†
White cell count (x 109/L)	5.6	5.7	5.3	0.209
Absolute neutrophil count	3	2.7	3.01	0.254
Hematocrit	32.3	31.7	34.9	0.0006†
Platelets (x109/L)	311	326	260	0.104
Cluster of differentiation (CD4)	427	447	405	0.556
Sodium (mmol/l)	140	140	141	0.08
Potassium (mmol/l)	4.2	4.2	4.3	0.122
Chloride (mmol/l)	104	104	104.5	0.221
Bicarbonate	19.5	20	19	0.538
Urea (mmol/l)	2.9	2.7	3.1	0.213
Creatinine (micromol/l)	53	49	68	<0.0001†
Total bilirubin (IU/L)	11	8.2	11	0.016†
Direct bilirubin (IU/L)	3.9	3.4	4.8	0.017†
Alanine Transaminase (IU/L)	27	27.5	27	0.407
Aspartate transaminase (IU/L)	36.1	39	33.3	0.028†
Alkaline phosphatase (IU/L)	155	167	107.3	<0.0001†
Total Albumin (g/L)	39.3	39	40.6	0.109
Total Protein (g/L)	80.9	81	80	0.9

* p-value from Mann Whitney U test; †": statistical significance; AYALHIV: adolescents and young adults living with HIV; HIV: human immunodeficiency virus; KBTH: Korle Bu Teaching Hospital

**Viral load:** approximately 78% (196/250) of AYALHIV had viral suppression (viral load less than 1000 cp/ml). Of those who had viral suppression, 91% (178/196) had viral loads less than 20 cp/ml. Among the adolescents, 75.6% (130/172) had viral suppression, with 84.6% (66/78) having viral suppression among the young adults.

## Discussion

This study highlighted the clinico-epidemiological characteristics of 252 AYALHIV. The majority of them were female (56.7%), adolescents (68%), had HIV type I infection only (85.7%), and were on first-line ART (NNRTI-based regimen) (88.1%). Fifteen percent of AYALHIV had both parents dead. Almost 38% had no mothers, while 33.7% had no fathers. About 88% of AYALHIV acquired HIV through MTCT with 10.7% acquiring it via unprotected sex. Almost eighty percent of AYALHIV had achieved viral suppression. Of those who achieved viral suppression, over 90% of them had viral loads to be less than 20 copies/ml. More than half of AYALHIV in this study were female. This is similar to the AYALHIV in Kenya [18], and Ghana [19], where females also form the majority. Also, the majority of the AYALHIV in this study were adolescents. This observation is however, expected since there is increased survival of perinatally-infected children due to the widespread availability of ART. However, other studies observed that the majority of their AYALHIV were young adults aged 20-24 years [20,21]. Different studies have suggested various modes of transmission of HIV among adolescents and young adults [18,22]. Maternal transmission was responsible for almost 90% of the HIV infection among AYALHIV in this study. Among the adolescents, the MTCT rate was 98% whilst among the young adults, it was 66.3%. These findings are as a result of missed opportunities to effectively implement prevention of mother-to-child transmission (PMTCT) interventions in Ghana at the time of conception of these children [23].

Over 487,752 antenatal care (ANC) clients were not tested for HIV from 2011 to 2013 across all the regions in Ghana at the time as PMTCT activities were now being scaled up [23]. These gaps highlighted in the primary care for maternal and child health care are likely to be responsible for the high rates of MTCT among our patients. However, over 30% of young adults acquire it through unprotected sexual intercourse. These findings are similar to that of another study in low/middle-income countries (LMIC) [18]. However, it was observed that 12% of AYALHIV most likely acquired the infection through MTCT, whilst about three-quarters of them acquired it sexually [18]. This difference can be explained by the fact that the majority of the participants in that study were older adolescents and young adults who had already had sexual debut. With this disproportionate rate of female infection, coupled with the high rate of MTCT, as well as the high pregnancy rate among adolescents in Ghana, the risk of MTCT is further increased among AYALHIV [24,25]. It has been noticed that pregnant adolescents living with HIV have poorer PMTCT outcomes. This can contribute to the increased prevalence of HIV among HIV-exposed infants who would survive into adolescence [26]. Again, AYALHIV can be a source of new infection to the uninfected population. There is a need to encourage HIV testing and diagnosis among the youth, and also strengthen sexual and reproductive health education. Future HIV programs should target adolescents and PMTCT.

About 22% of AYALHIV in this study were not virally suppressed. Other studies in sub-Saharan Africa reported similar percentages, 20%- 26% [27-29]. This could be due to unawareness of HIV status, inadequate understanding of the infection, increased pill burden, stigma (for those in boarding institutions), or lack of social support. Further studies are needed to investigate factors responsible for this high prevalence of non-suppression among AYALHIV and to identify strategies to enhance viral suppression. The prevalence of viral non-suppression reported by Umar *et al*. was however almost two times what was found in this study. All the study participants were aware of their HIV status, with almost 20% of them being non-adherent [30]. Thirty-eight percent (38%) and thirty-four percent (34%) of AYALHIV in this study had lost their mothers and fathers, respectively. The loss of parents causes a lot of changes in their lives. Health care, nutrition, education, financial support, and accommodation of AYALHIV are affected [31]. This increases the risk for early sexual debut, unprotected sexual activity, and pregnancy, owing to lack of parental control, poverty, and/or psychological distress [31]. Caregivers of these orphans, also struggle to cope with the demands of taking care of these AYALHIV.

Some battle with challenges such as job insecurities, and keeping up with hospital appointments for their wards [32]. Future interventions should include caregivers of these AYALHIV, as they play a very vital role in their overall management and development. These AYALHIV continue to experience risky health vulnerabilities and a global agenda to protect them must be instituted for maximum impact of sustainable development goals (SDGs) aligned provisions on their health. There is a need to focus on developing targeted programme interventions for this unique group to combat poverty and hunger in alignment with SDGs 1 and 2. Expanding interventions to ensure employment for these AYALHIV or for a caregiver and ensuring caregiver is healthy is essential to achieving SDGs 3 and 8. Thus, there must be efficient and effective planning by national HIV control programs to implement such targeted interventions to support access to available services for AYALHIV.

**Limitations:** this study is purely descriptive, and thus inferences could not be made. Due to the nature of this study, HIV service delivery challenges encountered by AYALHIV could not be ascertained, which would have been useful additional information for implementers and other stakeholders.

## Conclusion

The AYALHIV population is growing, with the majority of them acquiring the infection through vertical transmission. About two-fifths of them had lost at least one parent, which makes them vulnerable to socio-economic and psychological challenges. Programmatic interventions should focus on this vulnerable group to avert the issue of them becoming the fulcrum of the HIV epidemic and also have the needed support to be mentally stable. Data must be strengthened as a primary driver of transformation among AYALHIV since it is critical to ensure fairness and enablement among them.

### What is known about this topic

- *The booming population of adolescents and young adults living with HIV (AYALHIV) in sub-Saharan Africa;*
- *Inadequate existing human immunodeficiency virus intervention programs for AYALHIV;*
- *There are advocacies for AYALHIV-centered interventions to provide a holistic approach to their care*.

### What this study adds

- *This study provides baseline data that can be used by human immunodeficiency virus programs, policymakers, and other implementers especially in West Africa to plan and design AYALHIV-focused interventions;*
- *The study also highlights the need for paying critical attention to human immunodeficiency virus transmission among this vulnerable group as they could become the fulcrum of the epidemic*.
